# Stability of percutaneously implanted markers for lung stereotactic radiotherapy

**DOI:** 10.1120/jacmp.v14i5.4337

**Published:** 2013-09-06

**Authors:** Gitte F. Persson, Mirjana Josipovic, Peter von der Recke, Marianne C. Aznar, Trine Juhler‐Nøttrup, Per Munck af Rosenschöld, Stine Korreman, and Lena Specht

**Affiliations:** ^1^ Department of Radiation Oncology Copenhagen University Hospital Rigshospitalet Copenhagen Denmark; ^2^ Department of Oncology Copenhagen University Hospital Rigshospitalet Copenhagen Denmark; ^3^ Department of Radiology Copenhagen University Hospital Rigshospitalet Copenhagen Denmark; ^4^ Faculty of Science Niels Bohr Institute University of Copenhagen Copenhagen Denmark; ^5^ Department of Oncology Copenhagen University Hospital Herlev Hospital Herlev Denmark; ^6^ Department of Science Systems and Models Roskilde University Roskilde Denmark; ^7^ Faculty of Medical Sciences University of Copenhagen Copenhagen Denmark

**Keywords:** fiducial markers, lung tumors, image‐guidance, stability, stereotactic body radiotherapy

## Abstract

The purpose of this study was to evaluate the stability of complex markers implanted into lung tumors throughout a course of stereotactic body radiotherapy (SBRT). Fifteen patients referred for lung SBRT were prospectively included. Radio‐opaque markers were implanted percutaneously, guided by computed tomography (CT). Deep inspiration breath‐hold CT scans (BHCT) were acquired at planning and on three treatment days. The treatment days' BHCTs were registered to the planning BHCT. Intraobserver uncertainty in both tumor and marker registration was determined. Deviations in the difference between tumor and marker‐based image registrations of the BHCT scans during treatment quantified the marker stability. Marker position deviation relative to tumor position of less than 2 mm in all three dimensions was considered acceptable for treatment delivery precision. Intra‐observer uncertainties for image registration in the left‐right (LR), anterior‐posterior (AP), craniocaudal (CC) directions and three‐dimensional vector (3D) were 0.9 mm, 0.9 mm, 1.0 mm, and 1.1 mm (SD) for tumor registration and 0.3 mm, 0. 5 mm, 0.7 mm, and 0.7 mm (SD) for marker registration. Mean 3D differences for tumor registrations on all days were significantly larger than for 3D marker registrations (p = 0.007). Overall median differences between tumor and marker position were 0.0 mm (range ‐2.9 to 2.6 mm) in LR, 0.0 mm (‐1.8 to 1.5 mm) in AP, and ‐0.2 mm (‐2.6 to 2.8 mm) in CC directions. Four patients had deviations exceeding 2 mm in one or more registrations throughout the SBRT course. This is the first study to evaluate stability of complex markers implanted percutaneously into lung tumors for image guidance in SBRT. We conclude that the observed stability of marker position within the tumor indicates that complex markers can be used as surrogates for tumor position during a short course of SBRT as long as the uncertainties related to their position within the tumor are incorporated into the planning target volume.

PACS number: 87.57.nj, 87.55.ne

## I. INTRODUCTION

Lung tumors move during breathing, and tumor motion of more than 3 cm has been seen for tumors located near the diaphragmatic domes.^(1)^ Breathing‐adapted radiotherapy, such as respiratory beam gating or tumor tracking, compensates for tumor motion by only irradiating during a prespecified part of the breathing cycle or by letting the treatment beam follow the tumor motion. Both approaches rely on the ability to determine and predict the breathing‐related tumor motion based on an external or internal surrogate for tumor motion. The prediction of the correlation between the tumor and the surrogate positions must be verified throughout the treatment; the verification can be performed with repeated kV imaging of the tumor. However, not all lung tumors are well defined on kV images and, therefore, radio‐opaque markers implanted in or close to the tumor have been used as a surrogate for tumor position. Markers can be implanted percutaneously, guided by fluoroscopy or computed tomography (CT), or transbronchially, inserted in nearby small bronchi. The advantage of percutaneous implantation is the possibility to implant the marker directly into the tumor, assuring a good representation of tumor motion, but potentially at the cost of morbidity due to the risk of pneumothorax. Bronchoscopic insertion implies less morbidity, but insertion directly into the tumor is difficult and the stability of the markers has been questioned.^(^ ^2^ ^,^ ^3^ ^)^

When the position of the radio‐opaque markers is used as surrogate for tumor position during radiotherapy, it is important that the position of the markers relative to the tumor is stable throughout the course of radiotherapy. The stability of markers implanted for long‐course fractionated radiotherapy has previously been evaluated,^(4)^ but this is not the case for short‐course SBRT.

The aim of this study was to evaluate the stability of the position of complex radio‐opaque markers relative to the lung tumor throughout a course of stereotactic body radiotherapy (SBRT).

## II. MATERIALS AND METHODS

Fifteen patients were prospectively included between February 2009 and December 2010. Data collection was approved by the Danish Data Protection Agency (j.nr. 30–0484). The study was approved by the local ethical committee (jr.nr. H‐B‐2007–016) and reported to Clinicaltrials.gov (ClinicalTrials.gov Identifier: NCT00910546).

Study inclusion criteria were: referral for SBRT, non‐small cell lung cancer (NSCLC) or lung metastases, age > 40 years, WHO performance status < 2, and obtained signed informed consent. Exclusion criteria were: forced expiratory volume in 1 second (FEV1) > 0.51, centrally located tumor, or tumor located close to large vessels.

**Figure 1:**
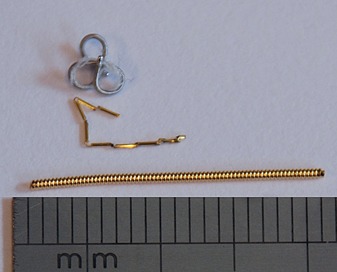
Photo showing the complex helical platinum marker (top), the Gold Anchor marker (middle), and the Visicoil gold marker (bottom).

All marker implantations were performed CT‐guided by the same radiologist, one week prior to treatment planning and two weeks prior to treatment start. Markers were changed during the study. The first five patients had implanted a Visicoil (IBA Dosimetry, Bartlett, TN) gold marker measuring 0.75 mm × 2 cm. Patient no. 6 had implanted a complex helical platinum coil (Boston Scientific, Natick, MA) measuring 20 mm restrained and 2 × 4 × 4 mm unrestrained. Patients 7 to 15 had implanted a Gold Anchor marker (Naslund Medical, Huddinge, Sweden) measuring 0.28 × 20 mm. For all patients only a single marker was implanted. All markers were complex in structure (Fig. 1).

The planning protocol for SBRT consisted of a PET/CT with contrast enhancement and, within two subsequent days, a 4D CT and a voluntary deep inspiration breath‐hold CT (BHCT) in the same session. Treatment planning was performed on the midventilation phase of the 4D CT, and a total dose of 45 Gy was delivered in 3 fractions within five to eight days. On all three treatment days a BHCT was acquired.

The BHCT scans were acquired during deep inspiration breath‐hold (10–20 seconds) without contrast enhancement, on a Siemens Sensation Open multislice CT scanner (Siemens Healthcare.

Erlangen, Germany) in a helical scan mode with a pitch of 1.2 and reconstructed with a pixel size of 0.98 mm × 0.98 mm and 3 mm plane separation.

For the purpose of the BHCT, the patient was asked to take a deep inspiration and hold it during the scan. The respiration signal was monitored with the Real‐time Position Management (RPM) system (Varian Medical Systems, Palo Alto, CA).

The BHCT scans were used for evaluation of marker stability, rather than the 4D CT, to avoid potential motion artifacts.^(5)^ To evaluate the stability of the marker's position, the planning BHCT $\left({\text{CT}}_{0}\right)$ was rigidly registered to the BHCT scans of the three treatment days $\left({\text{CT}}_{1‐3}\right)$ by a single observer. Both tumor and marker registrations were performed. A window setting of 700 Hounsfield units (HUs) with a level of ‐300 HU was used. Only translations were used for the image registration, since rotations can be difficult to assess with small spherical tumors. The difference between tumor and marker registration was used as a measure of the stability of the marker position within the tumor and was evaluated for the translational directions: left‐right (LR), anterior‐posterior (AP), and craniocaudal (CC). Furthermore, 3D vector displacement was calculated. A difference in marker position within the tumor of less than 2 mm in one of the three translational directions was considered acceptable for treatment precision.

To evaluate the extent of intra‐observer variation all the rigid registrations were performed twice. The standard deviation (SD) of the differences between the two registrations of the tumor was a measure of the intra‐observer variation in tumor registration, and the SD of the differences between the two registrations of the marker was a measure of the intra‐observer variation in marker registration.

The evaluation of the marker position deviation within the tumor was evaluated for all four combinations of differences between the tumor and the marker registration (i.e., the first tumor registration vs. the first and second marker registration, respectively, and the second tumor registration vs. the first and second marker registration, respectively). The mean of the four values was used in the analysis of the marker stability.

Parametric statistics with calculation of SD were applied for intra‐observer variation data as these were considered Gaussian‐distributed. The data concerning the stability were considered to be influenced by both the intra‐observer variation and the position deviation of the marker relative to the tumor, and therefore nonparametric statistics were applied.

## III. RESULTS

Patient characteristics are presented in Table 1. Only 14 patients were eligible for the stability evaluation. The marker of patient no. 2 disappeared between the planning scan and the treatment start. It had been placed in proximity to a bronchus and the patient had been reported coughing. It is hypothesized that the marker was coughed up, as a full‐body CT revealed no sign of the marker. Patient no. 8 was excluded after the first treatment session as the performance status had deteriorated and the treatment was terminated. For patients no. 5 and 15, scans from the second treatment sessions were missing because of technical issues. Altogether, data acquisitions were incomplete for four out of the 15 patients. In patient no. 6, the only patient with the complex helical platinum coil, the marker was implanted 1 cm below the tumor.

Intra‐observer variations in tumor registrations were 0.9 mm in LR, 0.9 mm in AP, 1.0 mm in CC direction, and 1.2 mm in 3D. Mean absolute deviations were 0.5 mm in LR, 0.6 mm in AP, 0.7 mm in CC direction, and 1.2 mm in 3D. For three patients (no. 3, 10, and 13), tumor match deviations exceeded 2 mm for one or two matches in one or two directions.

Intra‐observer variations marker registrations were 0.3 mm in LR, 0.5 mm in AP, 0.7 mm in CC direction, and 0.7 mm in 3D. Mean absolute deviations were 0.2 mm in LR, 0.3 mm in AP, 0.5 mm in CC, and 0.7 mm in 3D. No deviations in marker match exceeded 2 mm.

Intra‐observer variation for tumor registration was significantly larger than for marker registration (p = 0.007, paired Student's *t*‐test).

Overall median differences between tumor and marker position were 0.0 mm (range ‐2.9 to 2.6 mm) in LR, 0.0 mm (‐1.8 to 1.5 mm) in AP, and ‐0.2 mm (‐2.6 to 2.8 mm) in CC directions. The median absolute values for differences between tumor and marker positions were 0.3 mm (range 0–2.9 mm) in LR, 0.4 mm (0.1–1.8 mm) in AP, and 0.9 mm (0.1–2.8 mm) in CC directions. Median difference between tumor and marker position in 3D was 1.3 mm (range 0.4 to 4.3 mm).

**Figure 2:**
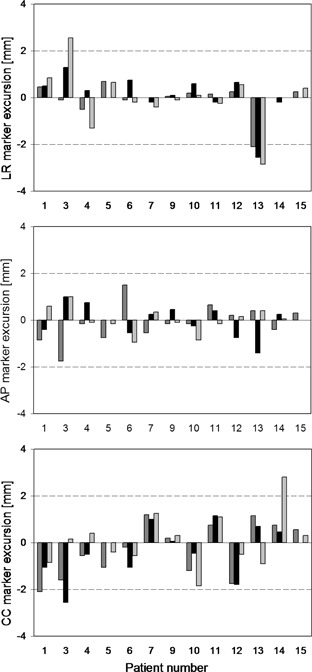
Position deviation of the marker relative to the tumor for all patients (${\text{BHCT}}_{0}$ as reference) at the first (▀), the second (▀), and the third (▀) treatment day.

Two patients (no. 1 and 14) had one single deviation between tumor and marker position exceeding 2 mm, both in the CC direction. Two patients (no. 3 and 13) had deviations in two or more registrations exceeding 2 mm; patient no. 13 had consistent deviations between tumor and marker position of 2.1 to 2.9 mm in the LR direction indicating a displacement of the marker between planning and treatment. Deviations for all patients are shown in Fig. 2.

**Table 1:** Patient characteristics

${\text{Age}}^{a}$		73 (60–87) years
*Gender*	Female / Male	7/8
*FEV1* ^a^		1.4 (0.7–3.4) L
*Diagnosis*	NSCLC	14
	Lung metastases	1
*Tumor Location*	Upper lobe	6
	Lower lobe	9
*Tumor Size*	Diameter^a^	3.6 (1.5–7.0) cm
	Volume^a^	$13.6\;(1.7–46.3)\;{\text{cm}}^{3}$
	Left–Right	0.2 (0.1–0.3) cm
*Tumor Motion* ^a^	Antero–Posterior	0.2 (0.1–0.4) cm
	Craniocaudal	0.6 (0.1–2.4) cm

a Median (range)

## IV. DISCUSSION

For nine of the evaluated 13 patients, all deviations between tumor and marker position were within 2 mm. For two of the remaining four patients only one measured marker position deviation exceeded the 2 mm threshold. The median (and the mean) deviations in all translational directions were close to zero, indicating a random uncertainty.

The analysis of intra‐observer variation was performed to have an order of magnitude for the general uncertainty of the registration procedure for marker and tumor registration. The intra‐observer variation of the tumor match was 0.9 to 1.0 mm (SD), depending on direction. Most of the observed differences between tumor and marker position were within the intra‐observer uncertainty for tumor registration.

Two patients (no. 3 and 13) had larger deviations in marker position. This observed variation was probably caused by poorly defined tumors which were difficult to match, as the intra‐observer variations in tumor match were largest for the same two patients. In patient no. 13, the tumor had poorly defined edges and was located close to the diaphragm, which resulted in 3 cm peak‐to‐peak motion in CC direction during free breathing. In patient no. 3, the tumor was embedded in fibrous tissue caused by earlier breast cancer irradiation. The marker was implanted in the periphery of the tumor, which may also have had an impact on the registration results. Smith et al.^(6)^ found that the closer to the tumor center the marker was implanted, the better it followed the tumor position, since tumor tissue is more rigid than surrounding lung parenchyma, while correlation between tumor motion and marker motion deteriorates as the distance from the marker to the tumor increases. Patient no. 6 had the marker implanted 1 cm inferior to the tumor; nevertheless, the marker stability in this patient did not stand out (see Fig. 2), possibly because the tumor was located in the lung apex where tumor and lung tissue motion is minimal. In our study, one of the patients presumably coughed up the marker. This problem is more often encountered with markers implanted by bronchoscope,^(^ ^2^ ^,^ ^7^ ^,^ ^8^ ^,^ ^9^ ^)^ and can be seen as an extreme deviation of marker position. This patient was excluded from the analysis, thus biasing the study. In a clinical setting using a marker‐based image‐guidance protocol, the patient would have had the marker replaced.

Intra‐observer variation of tumor registration was approximately twice the intra‐observer variation of marker registration. This difference was due to high radio‐opacity of the markers in the CT compared to the tumor, in addition to a well‐defined shape. Visicoil marker resulted in a higher degree of metal streaking artifacts in the CT images than the complex platinum coil and the Gold Anchor; however, these artifacts did not impact the visual definition of the marker shape. Lack of rotations in registration may also have affected the intra‐observer variation. Josipovic et al.^(10)^ showed that omitting rotational position corrections when applying soft tissue tumor registration as image guidance in SBRT, a systematic error of 1 mm (SD) was introduced

**Figure 3:**
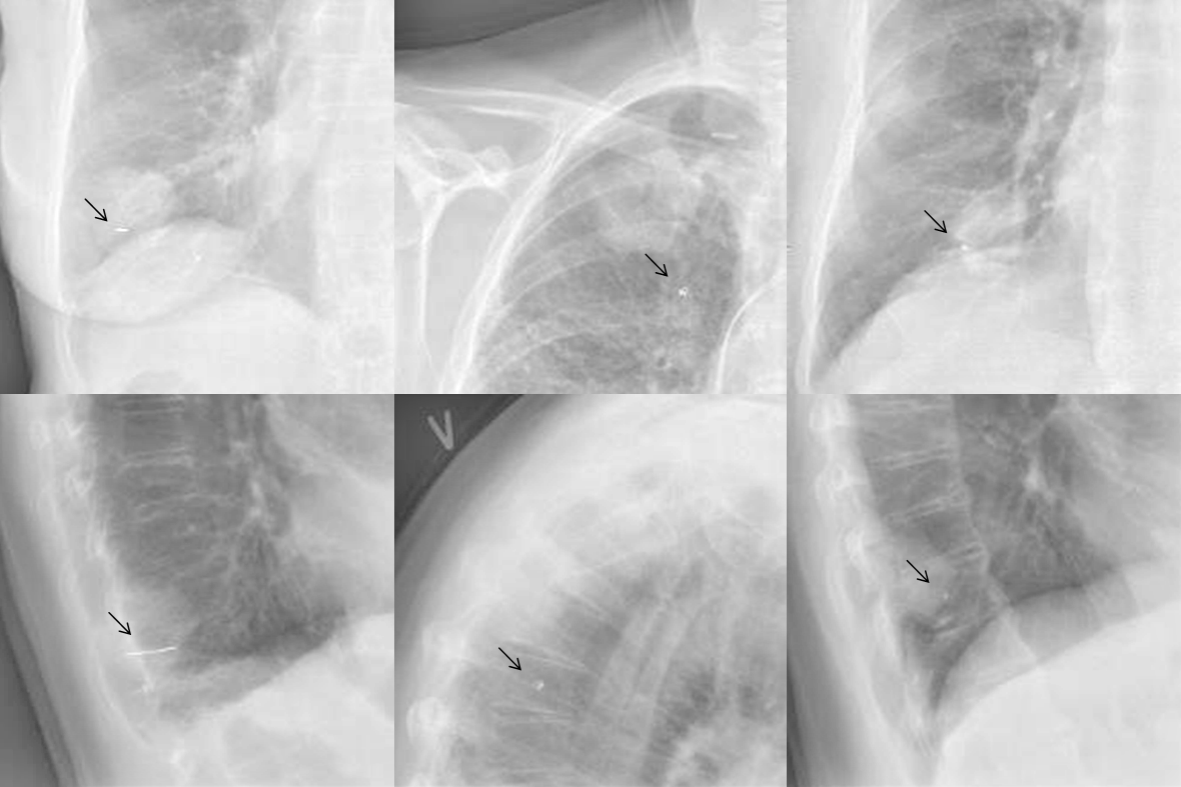
Chest X‐rays showing different marker types. Patient 1 had a 2 cm long helical gold marker (Visicoil) implanted in the tumor in the right inferior lung lobe (left). Patient 6 had the complex helical platinum marker (Boston Scientific) implanted in the tumor in right superior lung lobe (middle). Note that the marker was placed outside the tumor. The patient had a pacemaker. Patient 11 had the Gold Anchor marker implanted in the tumor in the right inferior lobe (right). The arrows indicate the position of the markers.

By visual evaluation of the thoracic orthogonal kV X‐rays acquired four hours after the marker implantation (Fig. 3), it was clear that the Visicoil marker provided the highest radio‐opacity. The two other markers provided equal radio‐opacity. However, the radio‐opacity of the Gold Anchor marker depended on the intratumoral folding of the marker.

Kupelian et al.^(4)^ included 15 patients for percutaneous implantation of Visicoil markers and eight patients for transbronchial implantation of metallic fiducial markers in lung tumors. The majority of the patients received long‐course radiotherapy (70 Gy/35 fractions). Expiration breath‐hold CT scans were acquired at planning and at a point during treatment or at follow‐up. The two scans were compared and the marker position deviation was estimated. For all patients, no translational deviations in marker position relative to the tumor larger than 5 mm were seen. As pointed out by the authors, tumor shrinkage of 34% ± 23% throughout the observation period impacted the results. The latter probably also explains that the reported deviations were significantly larger than in the present study with a much shorter treatment and observation time.

Roman et al.^(11)^ analyzed weekly 4D CT scans of seven patients with locally advanced NSCLC and bronchoscopically implanted gold coils receiving long‐course radiotherapy. They found interfractional marker‐to‐tumor centroid displacements of 2–3 mm both systematically and randomly in the three translational directions. They also found significant change in the displacements over time.

Van den Vort van Zyp et al.^(12)^ examined the stability of 111 smooth platinum markers implanted percutaneously in 44 lesions of 42 patients with lung tumors. BHCT were performed two or three times during a hypofractionated treatment course. They found a median marker displacement of 1.2 mm, but 12% of the markers moved more than 5 mm and 5% of the markers moved more than 10 mm. This displacement was also larger than the displacement found in our study and could be caused by the smooth structure of the markers compared to the complex or coiled structure of the markers in our study.

Schroeder et al.^(8)^ reported that coils are more stable than linear markers. Studies^2^, ^3^, ^9^ using spherical, cylindrical or linear markers reported marker migration rates of 10% to 69%, which was taken into account by the insertion of multiple markers. Also Hong et al.,^(13)^ in a retrospective study including 54 consecutive patients, found that coil markers were better retained than seed markers. In several other studies,^(^ ^2^ ^,^ ^3^ ^,^ ^7^ ^,^ ^8^ ^,^ ^9^ ^,^ ^14^ ^,^ ^15^ ^,^ ^16^ ^,^ ^17^ ^,^ ^18^ ^,^ ^19^ ^)^ multiple markers per tumor were implanted, as well. A single marker was implanted only in cases of small tumors, such as in our study. The advantage of multiple implanted markers is the possibility of using pattern recognition to check for tumor rotation, deformation, and displacements of markers during treatment. However, use of multiple markers also increases the risk of pneumothorax, compared to using a single marker.^12^, ^15^

A limitation of the present study is the limited number of patients included. However, a rigid imaging and data processing protocol enhances the validity of the study.

Use of different marker types in our study represented an unscheduled modification of the clinical protocol. The marker types were changed during the study by request of the radiologist.

The gold Visicoil marker gave the best radio‐opacity in kV imaging, but the implantation needle's outer diameter was 1.27 mm and resulted in two cases (patients no. 4 and 5) of pneumothorax; therefore, the marker was changed to the Boston Scientific complex helical coil (Boston Scientific, Natick, MA) for patient no. 6. However, this marker was not preloaded into the implantation needle and the instrumentation was difficult. The Gold Anchor marker was preloaded and inserted through a thinner needle (outer diameter 0.53 mm) minimizing the risk of pneumothorax, although two of the nine patients implanted with this marker had a pneumothorax (patients no. 7 and 10). The thin needle however, was very flexible, making its steering challenging. In our opinion, the optimal marker for IGRT and insertion method is yet to be found.

In our study, as in other studies examining marker stability,^(^ ^2^ ^,^ ^12^ ^,^ ^16^ ^,^ ^19^ ^,^ ^20^ ^)^ CT scans were used for the evaluation. It is important to be aware that interpolations in CT imaging will inherently lead to intramodality uncertainty in imaging, hence compromising the precision of information in images. In a recent study by Oxnard et al.,^(21)^ the variability between tumor size measurements on repeated BHCT showed differences exceeding 2 mm for a third of the patients.

## V. CONCLUSIONS

Overall we conclude that the deviations of marker position in the tumor were within the magnitude of the intra‐observer variation of the registration procedure for the majority of patients, and that the intra‐observer uncertainty in marker registration was significantly smaller than in tumor registration. The observed stability of marker position within the tumor indicates that markers implanted in lung tumors can be used as surrogates for tumor position during a short course of SBRT as long as the uncertainties related to their position within the tumor are incorporated into the planning target volume.

## ACKNOWLEDGMENTS

The authors have received grants from The Danish Council for independent Research in Medical Sciences”, The Arvid Nilssons Foundation, and The Astrid Thaysens Foundation.
